# The effect of polymer and CaCl_2_ concentrations on the sulfasalazine release from alginate-*N,O*-carboxymethyl chitosan beads

**DOI:** 10.1186/2194-0517-2-10

**Published:** 2013-04-04

**Authors:** Moslem Tavakol, Ebrahim Vasheghani-Farahani, Sameereh Hashemi-Najafabadi

**Affiliations:** Biotechnology Group, Faculty of Chemical Engineering, Tarbiat Modares University, P.O. Box 14115–143, Tehran, Iran

**Keywords:** Alginate, *N*,*O*-carboxymethyl chitosan, Ionic gelation, Blending, Sulfasalazine, Experimental design, Colon-specific drug delivery

## Abstract

In this study, pH-sensitive blended polymeric beads were prepared by ionic gelation of mixed alginate and *N*,*O*-carboxymethyl chitosan (NOCC) solutions in aqueous media containing calcium chloride. To prepare drug-loaded beads, sulfasalazine (SA) as a model drug was added to the initial aqueous polymer solution. These beads were characterized and evaluated *in vitro* as potential carriers for colon-specific drug delivery. A 3^2^ full factorial experimental design was employed to evaluate the effect of polymer and CaCl_2_ concentrations on swelling and drug release behavior of the beads in simulated gastrointestinal tract fluid. It was found that the rate of swelling and drug release decreased significantly with increasing polymer and CaCl_2_ concentrations, but polymer concentration was more effective than CaCl_2_ concentration. The beads prepared using 4.5% polymer concentration and 4% CaCl_2_ concentration retained approximately 60% of the loaded drug before approaching the simulated colonic fluid. Based on the results, the alginate-NOCC beads prepared with high polymer concentration could be potentially suitable polymeric carriers for colon-specific delivery of SA.

**Electronic supplementary material:**

The online version of this article (doi:10.1186/2194-0517-2-10) contains supplementary material, which is available to authorized users.

## Introduction

The use of polysaccharides in the formulation of colon-specific drug delivery carriers has gained increasing interest lately (Bajpai and Sonkusley 2002; Mahkam 2010; Mladenovska et al. 2007; Prabhu et al. 2008; Saboktakin et al. 2011; Tavakol et al. 2009). Micro- and nanoparticles prepared from some polysaccharides are attractive carriers for colon-specific drug delivery due to their favorite properties such as pH-sensitive swelling behavior, stability in the upper portion of the gastrointestinal tract, and suitable degradability by specific colonic enzymes (Assaad et al. 2011; Kim et al. 2012; Liu et al. 2007a; Sinha and Kumria 2001; Tavakol et al. 2009; Vandamme et al. 2002).

*N*,*O*-carboxymethyl chitosan (NOCC) is a chitosan derivative bearing a carboxymethyl substituent at some of the amino and primary hydroxyl sites of the glucosamine units of the chitosan structure. Biodegradability, biocompatibility, excellent water solubility, gel formation ability, and amphoteric polyelectrolyte characteristics make this material suitable for biomedical applications (Dolatabadi-Farahani et al. 2006; Fan et al. 2006; Lin et al. 2005; Tavakol et al. 2009; Upadhyaya et al. 2013; Zhang et al. 2004). Physically cross-linked carboxymethyl chitosan beads can be prepared by the dropping of aqueous low molecular weight (MW) carboxymethyl chitosan solution into CaCl_2_ solution (Liu et al. 2007b).

Alginate is a polyanionic copolymer of mannuronic and guluronic acid residues. Physically cross-linked Ca-alginate microparticles have been extensively studied as a potential carrier for oral drug delivery (Bajpai and Sharma 2004; Murata et al. 1993; Pasparakis and Bouropoulos 2006; Zhu et al. 2011). This system has major limitations such as rapid drug release caused by physical instability and high solubility of Ca-alginate beads in neutral and weak alkali media (George and Abraham 2006; Ma et al. 2010; Tavakol et al. 2009; Xing et al. 2003). To overcome these limitations, various approaches have been examined for the preparation of modified beads by blending and/or coating through polyelectrolyte complexation with polymers such as chitosan and chitosan derivatives (Chen et al. 2004; El-Sherbiny 2010; El-Sherbiny et al. 2010; Gong et al. 2011; Jayant et al. 2009; Lin et al. 2005; Meng et al. 2011; Mladenovska et al. 2007; Pasparakis and Bouropoulos 2006; Tavakol et al. 2009; Vandenberg et al. 2001; Zhu et al. 2011).

Lin et al. (2005) prepared a complex of alginate blended with NOCC by ionic gelation in Ca^2+^ solution. These beads demonstrated excellent pH sensitivity and could be a suitable polymeric carrier for site-specific bioactive protein drug delivery in the intestine. They used one-factor-at-a-time method to investigate the effect of polymer concentration and alginate/NOCC ratio on the properties of the beads, which are not useful in investigating interactions between factors. El-Sherbiny et al. (2010) prepared a new pH-sensitive hydrogel containing calcium-cross-linked blend of alginate and methacrylic (or acrylic) acid-grafted carboxymethyl chitosan. These beads showed high swelling degree and drug release percentage in simulated gastric fluid. To overcome these shortcomings, the beads were coated with poly(-chitosan copolymer (El-Sherbiny ethylene glycol)-*g*^2010^). This modification resulted in minimizing the swelling degree and loss of protein drug in the gastric fluid and preferably releasing the drug mostly in the intestine (El-Sherbiny 2010).

In our recent study, blended polymeric beads of alginate and NOCC were prepared and then coated by chitosan (Tavakol et al. 2009). The effect of coating as well as drying procedure on the properties of the beads, prepared at constant polymer and CaCl_2_ concentrations, were evaluated. It was found that the rate of swelling and drug release decreased for air-dried and coated beads in comparison with freeze-dried and uncoated ones, respectively (Tavakol et al. 2009).

In the present study, a 3^2^ full factorial design was performed to investigate the effect of polymer and CaCl_2_ concentrations, their interaction on the morphology and swelling characteristics of alginate-NOCC beads, as well as sulfasalazine (SA) release from these carriers in simulated gastrointestinal fluid.

## Methods

### Materials

Chitosan (MW approximately 2 × 10^5^) with an 85% degree of deacetylation was provided from Sigma-Aldrich Corporation (St. Louis, MO, USA). Sodium alginate was obtained from BDH Laboratory (London, England, UK). Calcium chloride, monochloroacetic acid and isopropyl alcohol were purchased from Merck (Darmstadt, Germany). Sulfasalazine was obtained from Zhejlang Jiuzhou Pharmaceutical Co. Ltd (Zhejlang, China). NOCC was synthesized according to the literature (Chen et al. 2004) and characterized by the method described by Sugimoto et al. (1998). All the other used chemicals, solvents, and reagents were of analytical grade.

### Preparation of beads

Firstly, aqueous alginate and NOCC solutions, with concentrations of 1.5%, 3%, and 4.5% (*w*/*v*), were prepared separately. Next, equal volumes of these solutions were mixed to form a homogenous blend solution which was maintained for 5 h for the complete removal of bubbles. The final pH of the solution was found to be approximately 7.5 ± 0.1. Five milliliters of these solutions was dropped into a 30-ml gently stirred CaCl_2_ solution with distinct concentrations of 1%, 2.5%, and 4% (*w*/*v*) through a syringe needle (0.4 mm in diameter) at a dropping rate of 1.0 ml/min. The distance of the needle tip from the gelling solution surface was 10 cm. The prepared beads were allowed to harden in the calcium chloride solution for 30 min. These beads were filtered, washed with distilled water three times, and dried at 40°C for 24 h or freeze-dried. The freeze-dried beads were obtained through rapid freezing at −80°C, followed by drying in a freeze drier (Zirbus, Denmark).

To prepare drug-loaded beads, SA with a final concentration of 1% (*w*/*v*) was added to the initial aqueous alginate solution with continuous stirring, and the pH of the solution was adjusted to 7.5 by adding 2 M NaOH. This solution was used for the preparation of SA-loaded beads by the same procedure described for the preparation of unloaded counterparts.

### Characterization of beads

The shape and surface characteristics of the beads were investigated by optical microscopy. The diameter of the beads was determined using an optical microscope and digital micrometer, and the average values were taken for at least 25 beads.

### Drug content and encapsulation efficiency determination

Encapsulation efficiency (wt.%) was calculated from the difference between the amount of SA dissolved in aqueous polymer solution and that of SA released in gelation medium divided by the amount of SA dissolved in aqueous polymer solution. For this purpose, the concentration of SA in gelation and washing solution was determined spectrophotometrically at 359 nm. Drug content (wt.%) was determined as the ratio of encapsulated SA weight to the total weight of the dried beads. This was accomplished by immersion of drug-loaded beads in sodium phosphate buffer at pH 7.4. The total released drug after 24 h was determined spectrophotometrically and was considered as encapsulated SA.

### Swelling studies

The swelling characteristics of beads were determined by immersing them in dry state into conical flask containing 40 ml of release medium that were incubated at 37°C under shaking at 150 rpm. At first, the dry beads were swollen in 0.1-M HCl solution at pH 1.2 (simulated gastric fluid) for 2 h. Afterwards, the beads were transferred to a sodium phosphate buffer solution at pH 6.8 (simulated small intestinal fluid) and kept for 3 h. Subsequently, they were transferred to a sodium phosphate buffer solution at pH 7.4 (simulated colonic fluid) until complete dissolution was obtained. At specific time intervals, samples were taken out from the swelling medium and blotted with a piece of paper towel to absorb excess water on the surface. The degree of swelling, *Φ*(*τ*), at each time was calculated using the following expression:

1 $$\Phi \left(\tau \right)=\left(\Psi_{\tau }-\Psi_{0}\right)/\Psi_{0}$$

where *Ψ*_*τ*_ and *Ψ*_0_ are the sample weights at time *τ* and in the dry state, respectively. Each experiment was repeated three times.

### Drug release studies

The SA release from drug-loaded beads was studied using the same conditions as described in the swelling studies. At predetermined time intervals, 2 ml of samples were withdrawn from the dissolution medium and immediately replaced by the same volume of fresh medium. The amount of SA released from the beads was determined spectrophotometrically (UV–vis Varian Cary 50, Varian, Inc., Palo Alto, CA, USA) at 359 nm using previously calibrated standard curves at different pH values. To determine the release in 0.1-M HCl solution, the pH of the release medium was adjusted to 7.4 by adding NaOH, and the concentration of SA was determined from the calibration curve at this pH. Each experiment was repeated three times.

### Experimental design and statistical analysis

A full factorial design with two parameters at three levels, as shown in Table 1, was applied. The experiments were carried out in random order to avoid any systematic error in the experimental data. Each experiment was repeated three times. Statistical software, Design Expert 7 (Stat-Ease, Inc., Minneapolis, MN, USA) and Minitab 14 (Minitab, State College, PA, USA), were used to analyze the experimental data.

**Table 1:** **Full factorial experimental design levels of polymer and CaCl**_**2**_ **concentrations**

Experimental run	1	2	3	4	5	6	7	8	9
Polymer concentration (g/100 ml)	1.5	1.5	1.5	3.0	3.0	3.0	4.5	4.5	4.5
CaCl_2_ concentration (g/100 ml)	1.0	2.5	4.0	1.0	2.5	4.0	1.0	2.5	4.0

## Results and discussion

### Characterization of beads

In our previous study (Dolatabadi-Farahani et al. 2006), the synthesized NOCC was analyzed by proton nuclear magnetic resonance spectroscopy based on a method described in the literature (Sugimoto et al. 1998). The degree of substitution of the carboxymethyl groups on the amino and primary hydroxyl sites was approximately 20.3% and 19.2%, respectively.

As expected, NOCC or alginate beads formed upon dropwise addition of aqueous NOCC or alginate solution into CaCl_2_ solution, due to ionic cross-linking between the carboxylate ions (−COO^−^) on NOCC or alginate, established by Ca^2+^. Thus, after the dropping of mixed alginate-NOCC solution into calcium chloride solution, alginate entangled through the NOCC network and vice versa, resulting in the formation of interpenetrating polymeric network. Zhang et al. (2004) showed that the blend membranes of carboxymethyl chitosan-alginate are miscible in the ratio from 1:1 to 1:5 and exhibited good mechanical properties due to strong electrostatic force and hydrogen bonding between different groups of two polymers.

**Figure 1:**
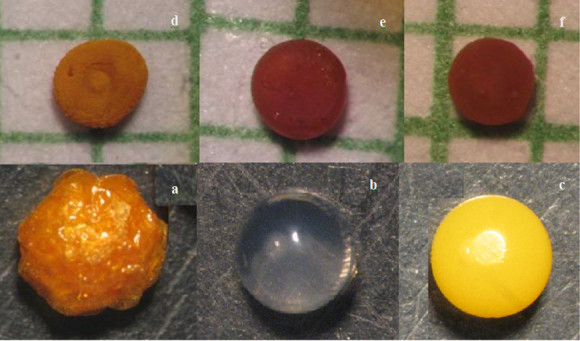
**The photographs of wet and dried alginate-NOCC beads taken under an optical microscope.** (**a**) Freeze-dried SA-loaded bead, (**b**) unloaded wet bead, (**c**) SA-loaded wet bead, and air dried SA-loaded beads prepared at different polymer concentrations (*w*/*v*): (**d**) 1.5%, (**e**) 3%, and (**f**) 4.5%.

The photographs of wet, freeze-dried, and air-dried beads, taken under an optical microscope, are shown in Figure 1. The diameters of the wet beads were 1.20 ± 0.10 mm independent of calcium chloride and polymer concentrations. After drying, the bead diameters slightly decreased from 0.70 ± 0.55 to 0.45 ± 0.60 mm with decreasing polymer concentration, but the effect of calcium chloride concentration was not significant. The wet beads were spherical in shape with a smooth surface. In the case of the beads prepared with 1.5% (*w*/*v*) polymer concentration, the spherical shape of the beads changed to an irregular shape with a collapsed center and some cracks on the surface (Figure 1c). The beads prepared with higher polymer concentration (3.0% and 4.5% (*w*/*v*)) remained almost spherical with a rather rough surface and compact structure.

### Swelling studies

**Figure 2:**
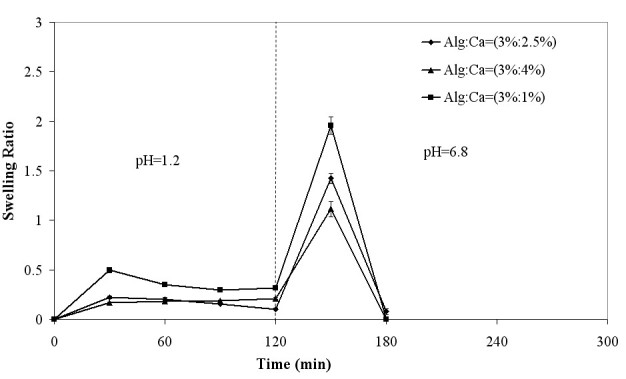
**Swelling behavior of alginate beads in simulated gastrointestinal fluid.**

**Figure 3:**
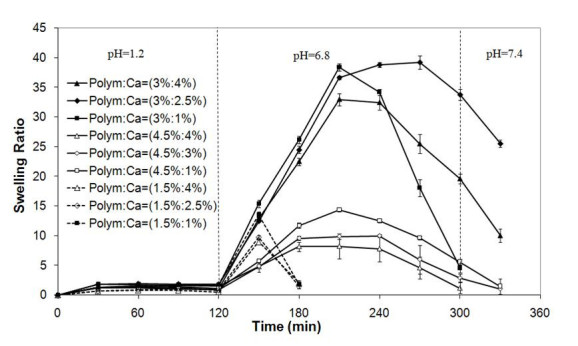
**Swelling behavior of alginate-NOCC beads in simulated gastrointestinal fluid.**

The swelling behavior of alginate and alginate-NOCC beads in simulated gastrointestinal fluid is shown in Figures 2 and 3, respectively. The swelling behavior of NOCC beads could not be studied due to the formation of very mechanically weak beads that lost their shape and were destroyed in the washing or drying steps.

At pH 1.2, the swelling of alginate and alginate-NOCC beads was hindered due to the formation of strong hydrogen bonds between -COOH and -OH groups of both polymer polar chains (Tavakol et al. 2009) and increased electrostatic attraction between protonated amine groups of NOCC and carboxyl groups of alginate in alginate-NOCC beads (Zhang et al. 2004).

At pH 6.8, alginate and alginate-NOCC beads began to swell noticeably due to the swelling force that resulted from the presence of counterions which neutralized the ionized carboxylic groups on alginate and NOCC and electrostatic repulsion between the ionized carboxylic groups (Lin et al. 2005; Tavakol et al. 2009). This phenomenon can also be related to ion-exchange between the Ca^2+^ ions in the hydrogel network and Na^+^ ions in the phosphate buffer solution (Bajpai and Tankhiwale 2006). Finally, the beads start to disintegrate, owing to the highly hydrated structure and almost complete removal of calcium ions (Bajpai and Tankhiwale 2006). The appearance of turbidity and observation of precipitate in the swelling medium, especially in the case of the beads prepared at higher calcium chloride concentration, are also indicative of the ion-exchange process.

At neutral and basic media, the swelling degree of alginate-NOCC beads, prepared at constant polymer concentration, decreased with increasing CaCl_2_ concentration (*p* < 0.05), due to increased cross-linking density of the network. At these media, the swelling and disintegration rate decreased significantly with increasing polymer concentration (*p* < 0.05). This may be related to increased elastic force that resulted from (1) hydrogen bonding between amine and hydroxyl groups of NOCC and alginate, (2) electrostatic attraction between ionized amine and carboxyl groups of NOCC and alginate, and (3) physical cross-linking as a result of polymer chain entanglements.

Comparing Figures 2 and 3, the disintegration rate of alginate-NOCC beads in PBS was significantly lower than that of alginate beads. This can be related to the presence of strong hydrogen bonds between the hydroxyl and amine groups of alginate and the NOCC and electrostatic attraction between the ionized amine and carboxyl groups of these polymers, which resist to disintegration of network.

### Drug release studies

**Figure 4:**
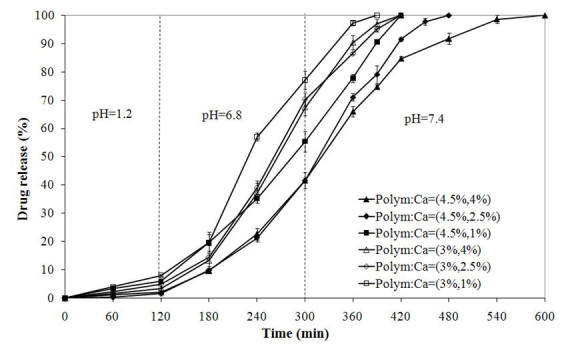
**Sulfasalazine release from alginate-NOCC beads in simulated gastrointestinal fluid.**

SA release from alginate-NOCC beads in simulated gastrointestinal fluid as a function of polymer and CaCl_2_ concentrations is shown in Figure 4. Drug release profiles indicate that the SA release from beads at pH 1.2 is relatively slow. This is due to the limited swelling degree of hydrogel network and solubility of SA at this pH. Subsequently, the SA release rate at pH 6.8 and 7.4 increased significantly (*p* < 0.05) in accordance with the swelling behavior of beads (Figure 3) and solubility of SA. The SA release rate decreased with increasing polymer and CaCl_2_ concentrations in accordance with the swelling behavior of the beads.

Analysis of variance, performed by Minitab 14 software, for the effect of polymer and CaCl_2_ concentrations on the total drug that remained in the beads before exposing them to the simulated colonic tract fluid (during the initial 5 h of release time) is given in Table 2. This analysis shows that the effects of both factors and their quadratic terms were significant, without having a significant interaction effect.

**Table 2:** **Analysis of variance of responses**

Source	SS	DF	MS	***F*** value	***p*** value	
Model	9,493.77	4	2,373.44	144.36	<0.0001	Significant
A (polymer concentration)	8,665.86	1	8,665.86	527.10	<0.0001	
B (CaCl_2_ concentration)	672.71	1	672.71	40.92	<0.0001	
A^2^	72.27	1	72.27	4.40	0.0477	
B^2^	82.93	1	82.93	5.04	0.0351	
Residual	361.69	22	16.44			
Lack of fit	43.67	4	10.92	0.62	0.6554	Not significant
Pure error	318.02	18	17.67			
Total	9,855.46	26				

This analysis of responses is in terms of total drug that remained in the beads (before exposure to the simulated colonic tract fluid) SS, sum of squares; DF, degree of freedom; MS, mean square; *F* value, factor effect value; *p* value, probability value.

**Figure 5:**
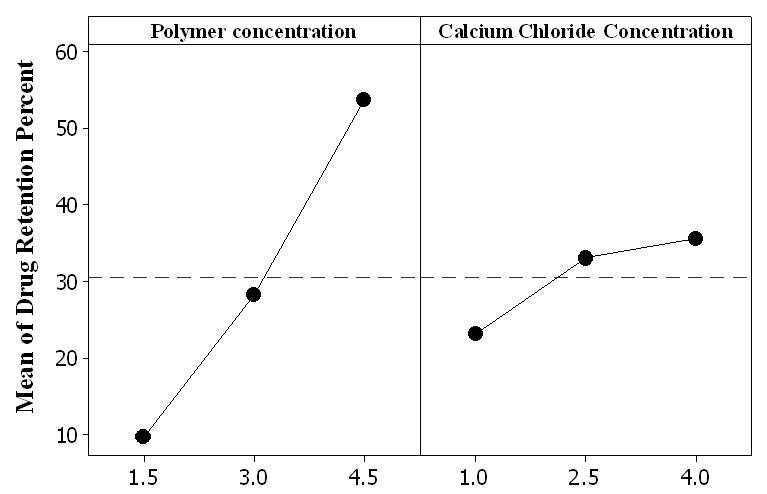
**Main effect plot for drug retention within carriers before exposing them to simulated colonic fluid.**

**Figure 6:**
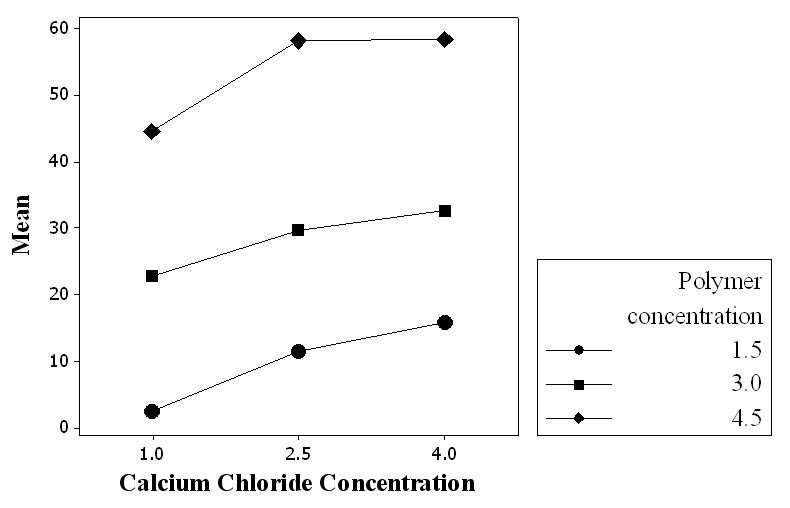
**Interaction plot for drug retention within carriers before exposing them to simulated colonic fluid.**

As shown in the main effect plot presented in Figure 5, the effect of polymer concentration on the retention of drug within the beads was higher than that of CaCl_2_ concentration. According to the interaction plot of these factors presented in Figure 6, the effect of CaCl_2_ concentration increasing from 2.5% to 4% became smaller at higher polymer concentrations. This can be related to the formation of densely cross-linked polymeric layer on the surface of droplets which resists to Ca^2+^ diffusion into the beads' core, leading to the formation of beads with unreacted or partially reacted core with smaller resistance to swelling and drug release.

As shown in Figure 4, the alginate-NOCC beads prepared at the polymer concentration of 4.5% and CaCl_2_ concentration of 4% or 2.5% retained approximately 60% of loaded drug before exposure to the simulated colonic fluid. This is a promising property for the application of optimized alginate-*N*,*O*-carboxymethyl chitosan gel beads as colon-specific delivery system.

The swelling and drug release characteristics of the alginate-NOCC beads can be tuned by the modulation of polymer and CaCl_2_ concentrations. Therefore, the beads can be further evaluated for the release of drugs in the different segments of the gastrointestinal tract. This strategy is more convenient than the coating procedure used in a previous study (Tavakol et al. 2009).

## Conclusions

Calcium cross-linked alginate-NOCC beads prepared in the present study demonstrated distinct pH-sensitive swelling and drug release behavior. Sulfasalazine release rate was slow in acidic medium but increased at pH 6.8 and pH 7.4, which is in accordance with the swelling rate of the beads and SA solubility. The rate of SA release decreased with increasing polymer and CaCl_2_ concentrations, but polymer concentration was more effective. No burst effect was observed for SA release from these pH-sensitive carriers. It was previously shown that the drug release behavior of SA-loaded alginate-NOCC beads was improved by chitosan coating (Tavakol et al. 2009). Based on these results, a suitable polymeric carrier for colon-specific delivery of SA can be developed by either the increasing concentration of alginate-NOCC blend solution or chitosan coating.

## Authors’ information

MT is a PhD student. EVF is a professor and supervisor. SHN is an assistant professor and co-supervisor.

## Authors’ original submitted files for images

Below are the links to the authors’ original submitted files for images. Authors’ original file for figure 1 Authors’ original file for figure 2 Authors’ original file for figure 3 Authors’ original file for figure 4 Authors’ original file for figure 5 Authors’ original file for figure 6
