# Supplementary figures and images for: The effect of polymer and CaCl2 concentrations on the sulfasalazine release from alginate-N,O-carboxymethyl chitosan beads
Source: Prog Biomater. 2013 Apr 4;2:10. doi: 10.1186/2194-0517-2-10 (PMC5151116; doi:10.1186/2194-0517-2-10)

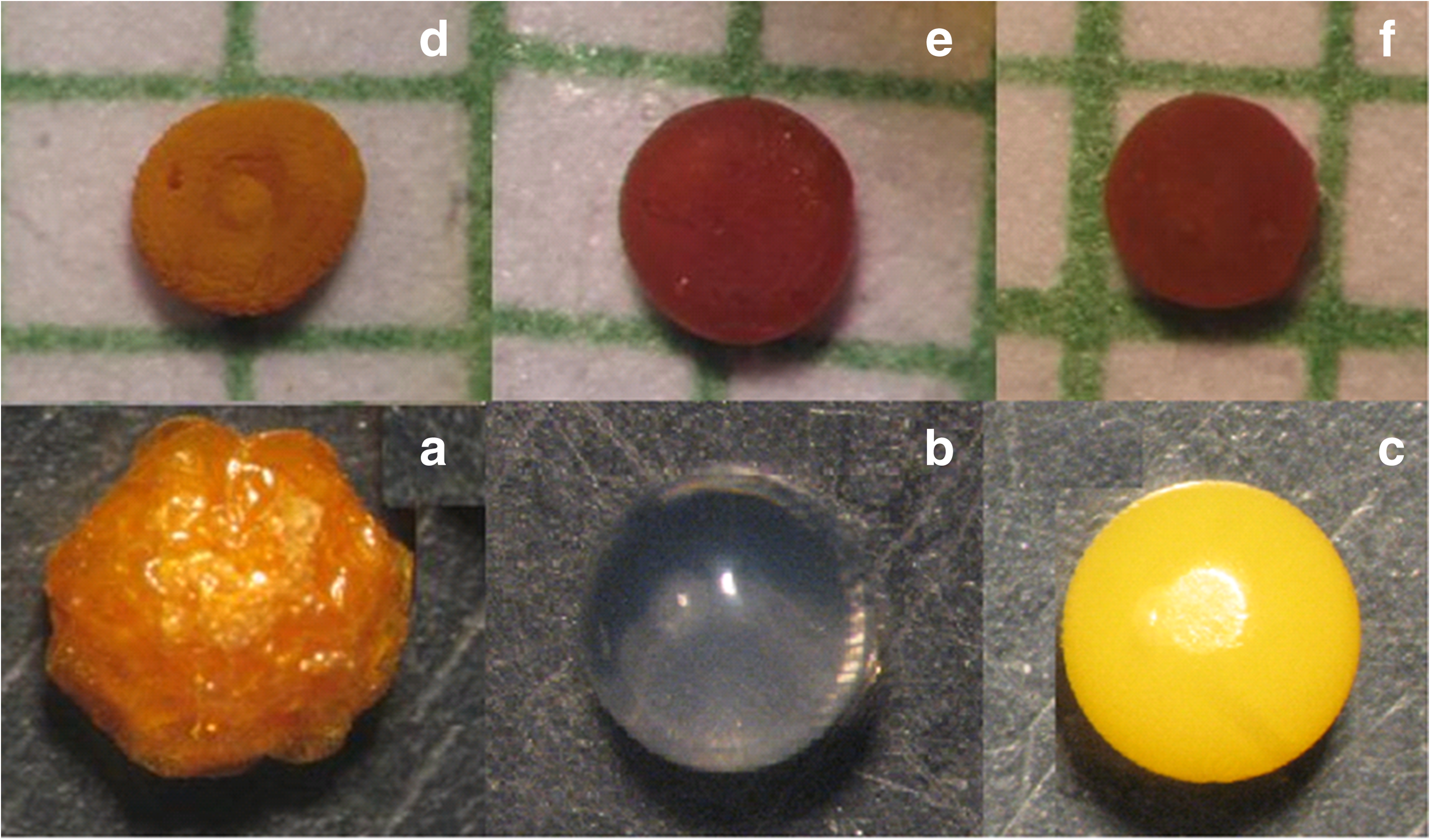

Supplement: Supplementary file 1 — Authors’ original file for figure 1 [file 40204_2012_11_MOESM1_ESM.tiff]

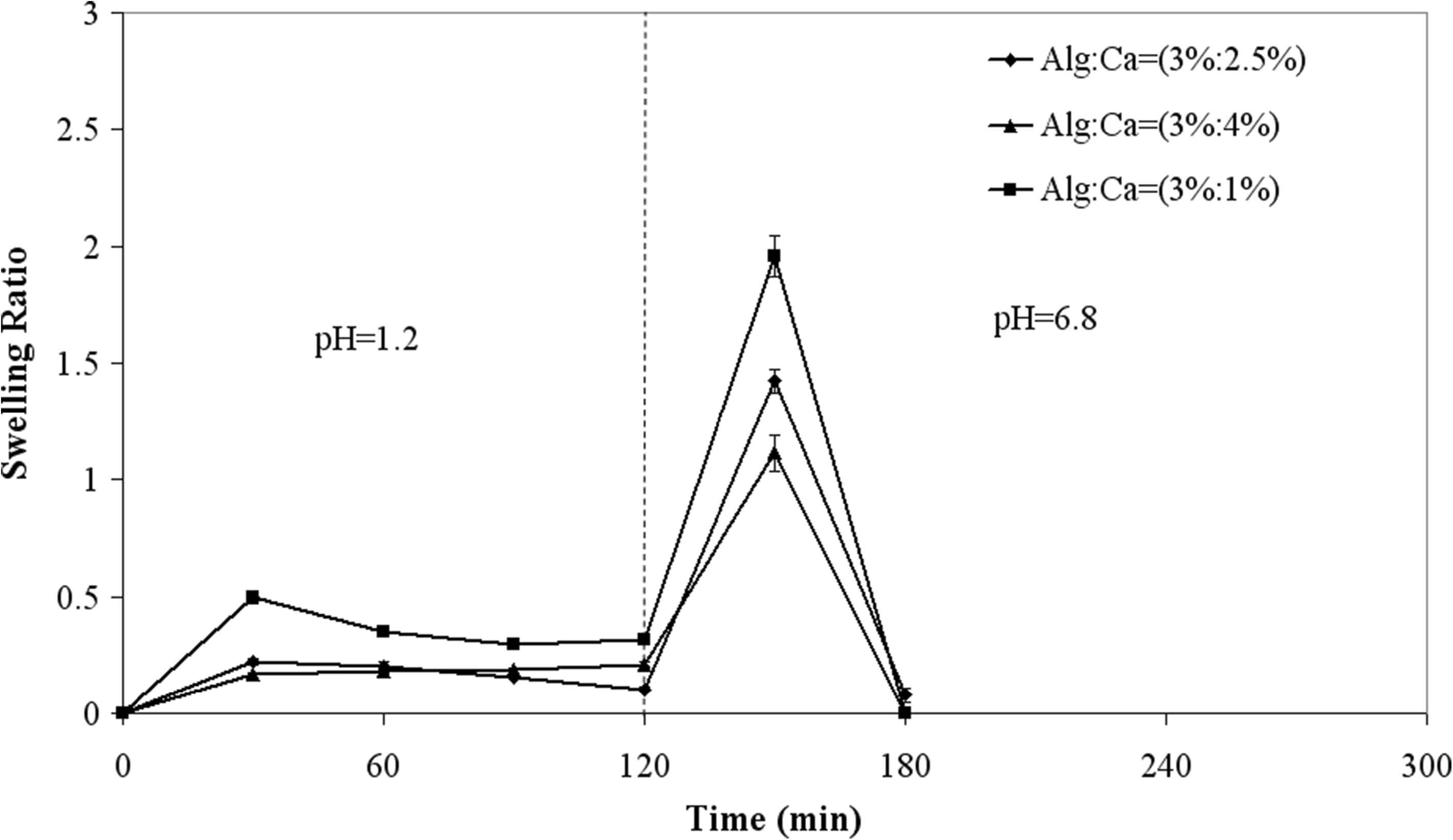

Supplement: Supplementary file 2 — Authors’ original file for figure 2 [file 40204_2012_11_MOESM2_ESM.tiff]

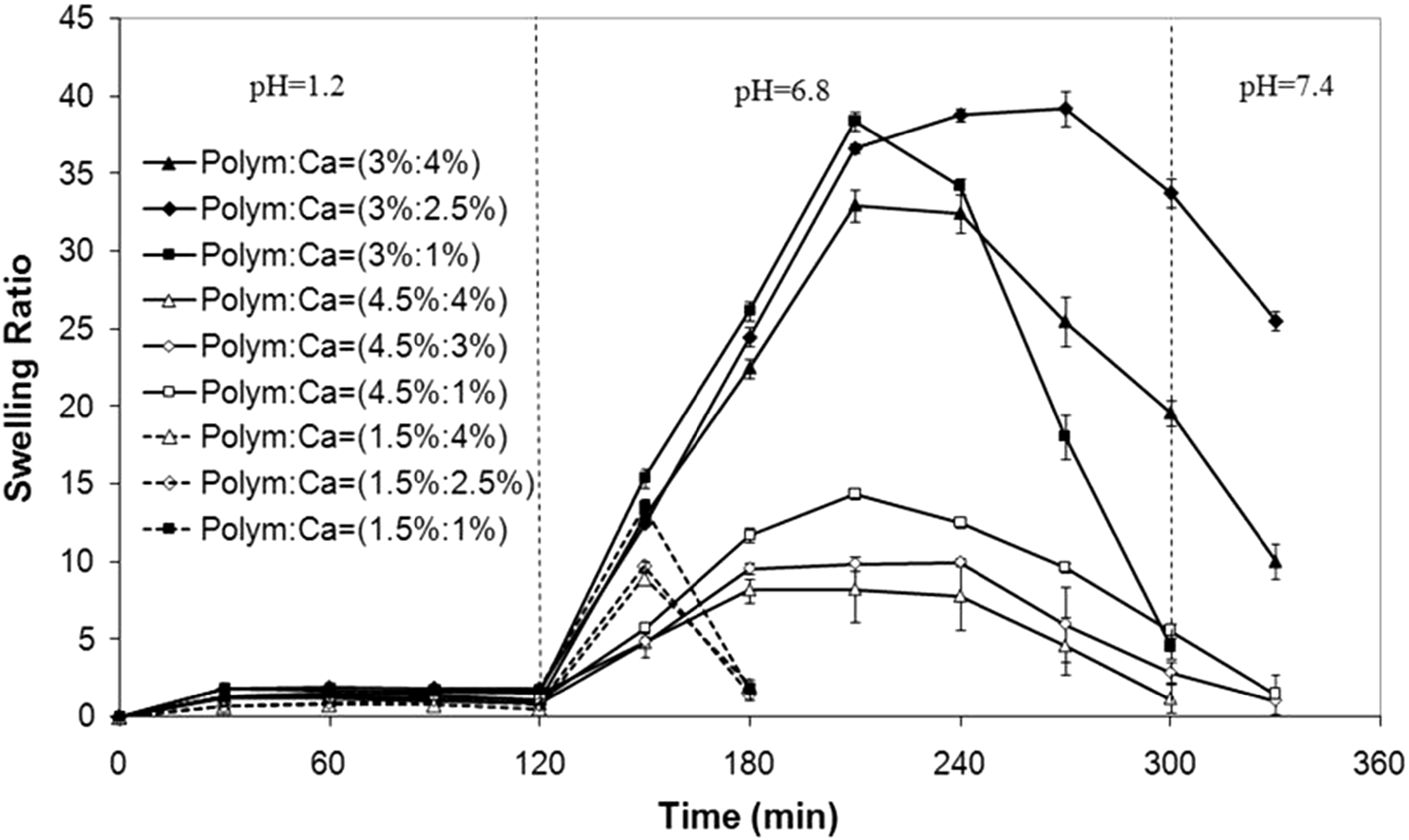

Supplement: Supplementary file 3 — Authors’ original file for figure 3 [file 40204_2012_11_MOESM3_ESM.tiff]

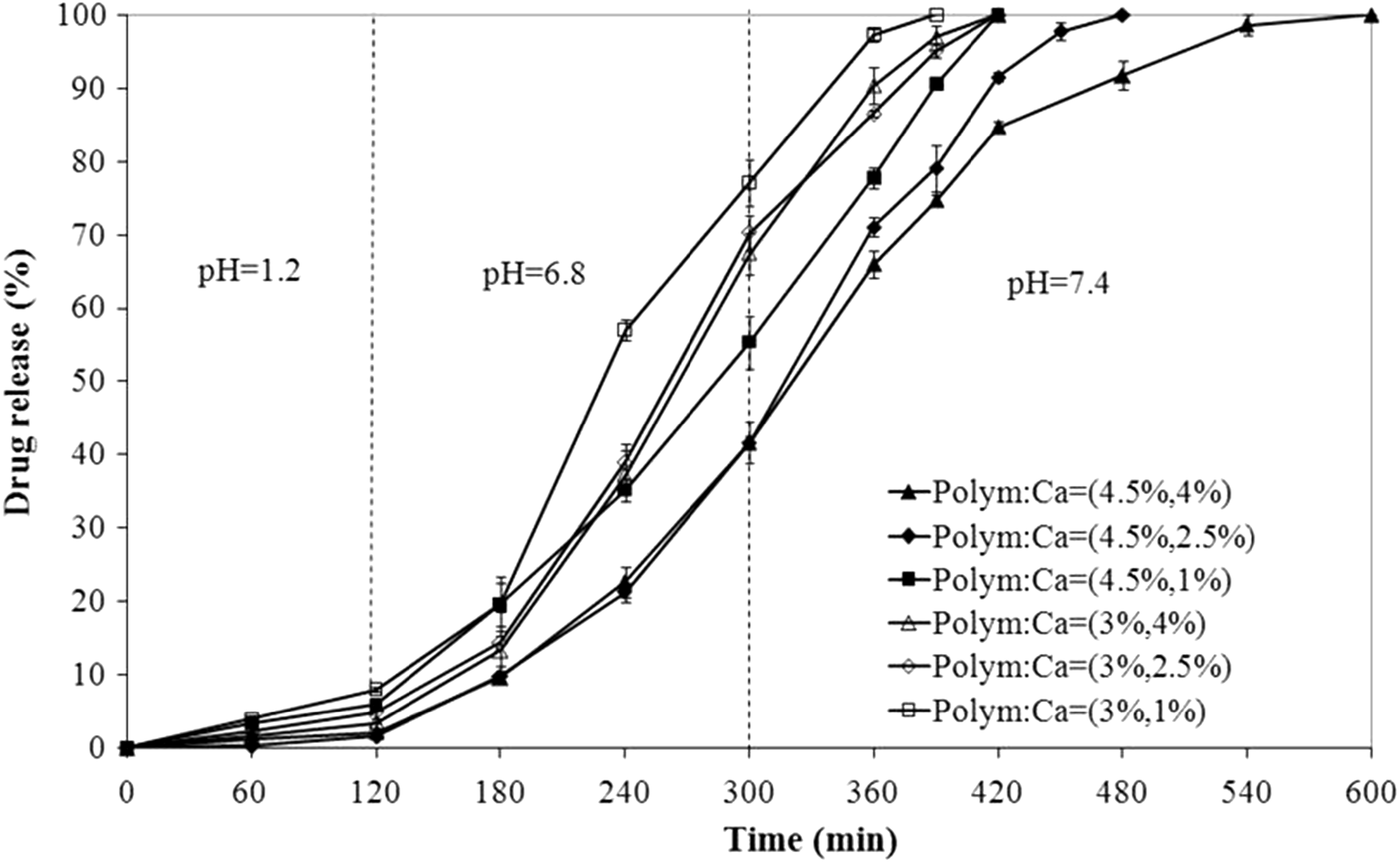

Supplement: Supplementary file 4 — Authors’ original file for figure 4 [file 40204_2012_11_MOESM4_ESM.tiff]

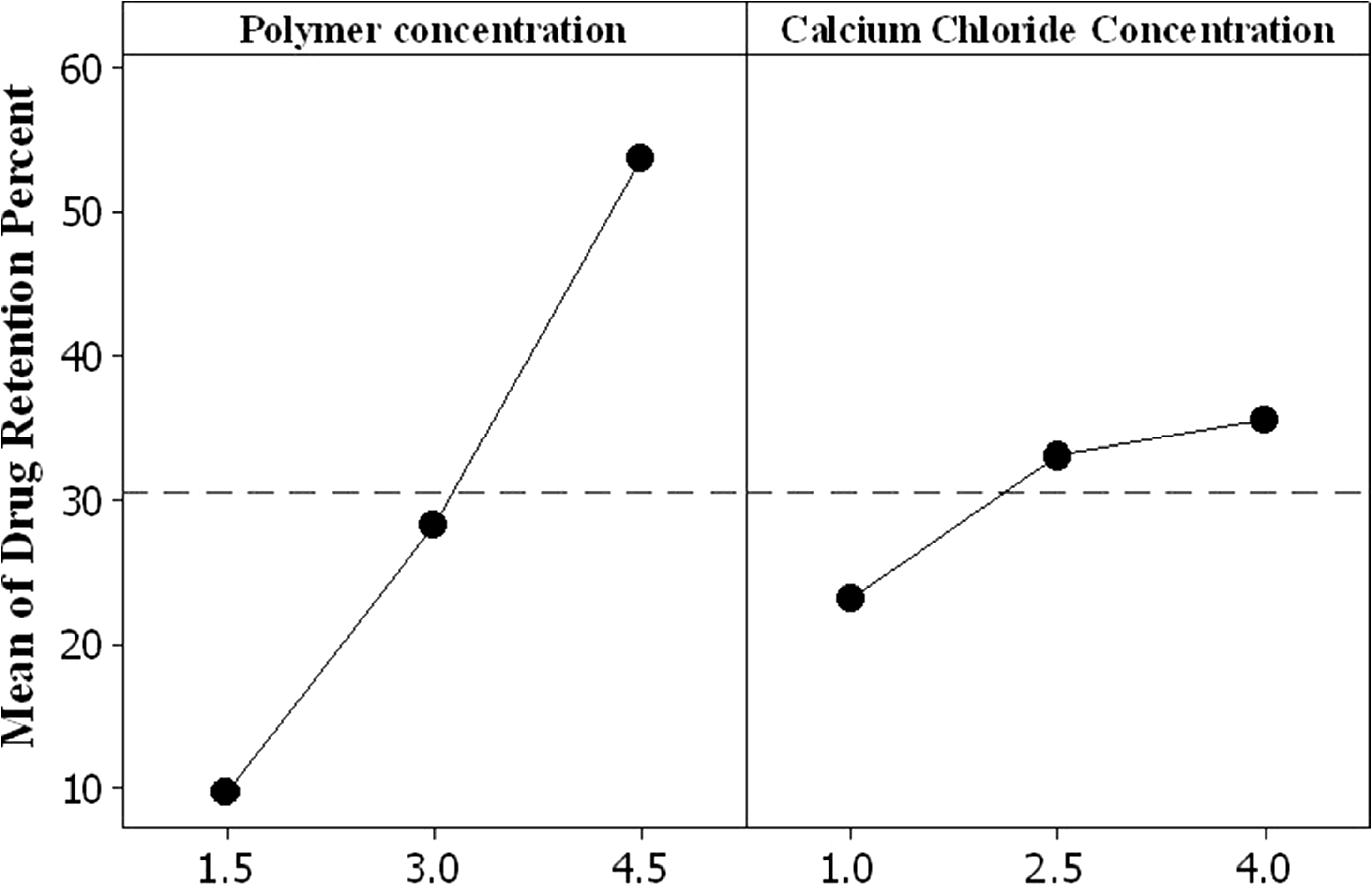

Supplement: Supplementary file 5 — Authors’ original file for figure 5 [file 40204_2012_11_MOESM5_ESM.tiff]

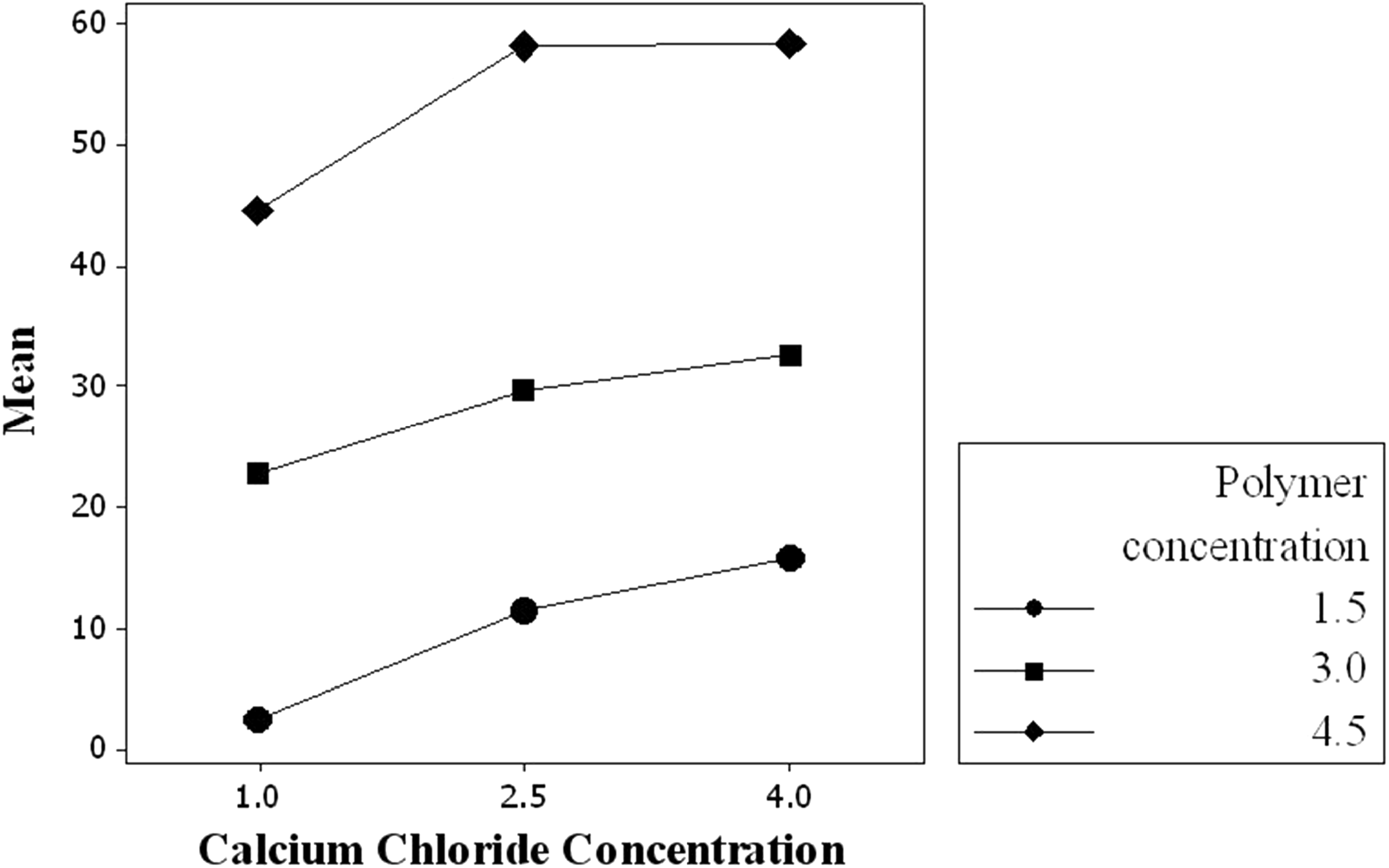

Supplement: Supplementary file 6 — Authors’ original file for figure 6 [file 40204_2012_11_MOESM6_ESM.tiff]
